# Geographical inequalities in skin-to-skin contact after birth among women in Somaliland: a multilevel and spatial analysis of the 2020 survey

**DOI:** 10.3389/fped.2026.1859038

**Published:** 2026-08-06

**Authors:** Abdiasis Aden Omer, Hamda Jama Yousuf, Mohamed Hussein Egeh, Ahmed Abdirahman Farah, Ali Ahmed Hussein, Fatima Nouh Ismail, Bashir Mohamed Mohamoud

**Affiliations:** 1 Ogaansho Research and Consultancy Center, Burao, Somaliland; 2 Department of Education, Faculty of Education, University of Burao, Burao, Somaliland; 3 School of Postgraduate Studies and Research, Abaarso Tech University, Burao, Somaliland

**Keywords:** DHS, multilevel analysis, neonatal care, skin-to-skin contact, Somaliland, spatial analysis

## Abstract

**Background:**

Skin-to-skin contact (SSC) after birth is a proven, low-cost intervention that improves neonatal survival and promotes early breastfeeding. This study aimed to assess the prevalence, determinants, and geographical inequalities of SSC after birth among women in Somaliland.

**Methods:**

A cross-sectional study was conducted using data from the 2020 Somaliland Demographic and Health Survey. A nationally representative sample of women aged 15–49 years who had a live birth within five years preceding the survey was included. Multilevel mixed-effects logistic regression analysis was employed to identify individual- and community-level factors associated with SSC. Model fitness was assessed using Akaike Information Criterion (AIC) and Bayesian Information Criterion (BIC). Spatial analysis was performed using R software to examine geographic variation, including hotspot analysis (Getis-Ord Gi*) and Local Moran's I to identify clustering patterns.

**Results:**

The prevalence of skin-to-skin contact after birth was 24.81%. Significant regional variation was observed, with higher prevalence in western regions and lower prevalence in eastern regions. In the multilevel analysis, women residing in Marodijeh (AOR = 0.36; 95% CI: 0.23–0.56), Sool (AOR = 0.48; 95% CI: 0.34–0.68), and Sanaag (AOR = 0.54; 95% CI: 0.34–0.86) had lower odds of practicing SSC compared to Awdal. At the individual level, wealth index, antenatal care utilization, place of delivery, pregnancy wantedness, and child size at birth were significantly associated with SSC. Women who delivered in health institutions had significantly higher odds of practicing SSC (AOR = 6.09; 95% CI: 4.39–8.45). Spatial analysis revealed significant clustering of low SSC practice in eastern regions, indicating clear geographic inequalities.

**Conclusion:**

The prevalence of skin-to-skin contact in Somaliland remains low, with substantial socioeconomic and geographic disparities. Both individual and community-level factors influence its practice, with institutional delivery emerging as the strongest predictor. Targeted interventions focusing on improving antenatal care utilization, promoting facility-based delivery, and addressing regional inequalities are essential to enhance the uptake of SSC and improve neonatal health outcomes.

## Background

According to the World Health Organization (WHO), immediate skin-to-skin contact (SSC) refers to placing the newborn, after drying, in a prone position directly on the mother's bare chest or abdomen immediately after birth or within the first hour of life, with uninterrupted contact maintained for at least one hour or until after the first breastfeeding, provided that both the mother and newborn are clinically stable. Immediate SSC is recommended as a standard component of essential newborn care because it promotes thermal regulation, cardiorespiratory stability, early initiation of breastfeeding, maternal–infant bonding, and improved neonatal survival, particularly in low-resource settings (1–4). Skin-to-skin contact (SSC) involves placing newborns (NB) on their mothers' bare abdomen or chest while they are naked and in a prone position, covered only by dry, previously heated blankets (5–8).

Placing the diapered baby on the mother's bare chest for daily skin-to-skin contact (SSC) has the potential to improve postpartum healing and reduce postnatal maternal mental health issues. Originally developed as an alternative to incubator care for preterm and low birthweight infants, the SSC method has been linked to even better infant outcomes, such as regulation and health. The mother benefits from daily SSC for preterm babies as well, including better bonding and longer breastfeeding initiation and duration. Additionally, daily SSC was linked to lower levels of maternal depressive symptoms in two studies involving preterm infants, and it was linked to lower levels of stress and anxiety during the first six postnatal months in another study. SSC is now recommended for healthy full-term infants during the initial postnatal hours and days for similar reasons (9).

Approximately 2.5 million newborns worldwide pass away in their first month of life, making up 47% of deaths among children under five. Even though the number of neonatal deaths decreased by 50% from 5 million in 1990 to 2.5 million in 2018, the decline in neonatal deaths has been slower than the overall decline in post-neonatal deaths among children under five. Childhood mortality has been linked to a number of factors. A suggested natural intervention for raising neonatal survival rates is SSC. In light of drying, wrapping, and postponing bathing, it is a part of thermal care. Every newborn should receive immediate newborn care following delivery, including thermal care, hygienic cord care, and early breastfeeding initiation, according to the World Health Organization (WHO). Thus, newborns exposed to SSC may have higher levels of physiological stability, thermoregulation, blood glucose, and early breastfeeding initiation. Additionally, because SSC improves bonding and caregiving behaviour toward the newborn and lowers maternal anxiety, it helps mothers with postpartum depression symptoms. By offering warmth, affection, stimulation, and defence against nosocomial sepsis, SSC can increase survival in prematurity. This can save lives in environments with limited resources because there is frequently insufficient knowledge or technology to handle complications (10). In Croatia, 98% of people have immediate skin-to-skin contact, compared to 1% in Tanzania. Skin-to-skin contact between the mother and child has become practically standard care in some nations, including Canada (81.0%), Australia (93.0%), Switzerland (95.4%), and Italy (80.4%). However, this is still rare in some nations, like Mexico (9.0%), India (14.5%), and Japan (20.0%). Skin-to-skin contact in teaching and non-teaching hospitals is approximately 59% and 53%, respectively, according to an Iranian study (11, 12).

Despite these established advantages, SSC adoption is still uneven throughout the world. According to a systematic review, prevalence was consistently lower in low- and middle-income countries, ranging from less than 10% in some African countries to over 95% in some parts of Europe. SSC prevalence in sub-Saharan Africa averages about 45%, although national estimates vary greatly due to variations in health system capacity and delivery methods. For example, prevalence has been reported as low as 12% in Nigeria. Similar low coverage has been reported outside of Africa in low- and middle-income settings, such as Bangladesh, highlighting the fact that suboptimal SSC uptake is not limited to a single geographic setting (13). Despite the evidence, skin-to-skin contact has not been widely adopted or implemented in SSA, and coverage has been low in most countries, ranging from 2% in Uganda to 10% in Nigeria and Ghana to 36% in The Gambia. Despite the WHO's recommendation for immediate or early skin-to-skin contact, it has been shown that mother and newborn separation occurs frequently in most situations. Determining the frequency of skin-to-skin contact is therefore necessary. A lack of nurses, a heavy workload, a lack of knowledge, time constraints, difficulty determining eligibility for skin-to-skin care, a lack of social support, a lack of guidelines, policies, and cultural norms were among the barriers mentioned in the few studies that looked into the reasons for the low uptake of skin-to-skin contact for newborns. According to a recent population-based cross-sectional study from The Gambia, skin-to-skin contact was determined by the place of delivery, residence, and timing of antenatal care (ANC) booking. However, there is a dearth of research on the factors that influence skin-to-skin contacts in SSA. The current study estimated the prevalence of skin-to-skin contact between mothers and their newborns as well as the predictors using nationally representative data from 17 sub-Saharan African nations (14).

In order to improve maternal and neonatal health outcomes in Somaliland, this study seeks to provide solid and nationally representative evidence to guide policies and strategies. The results help achieve the Sustainable Development Goals (SDGs) of the United Nations, especially SDG 3, which focuses on promoting well-being and guaranteeing healthy lives for everyone at all ages. The study specifically supports Target 3.8, which aims to achieve universal health coverage through improved access to essential maternal and newborn health services, and Target 3.2, which aims to lower neonatal mortality. This study guarantees a nationally representative analysis by using data from the 2020 Somaliland Demographic and Health Survey (SLDHS), which improves the findings' dependability and generalisability. To improve maternal and neonatal health outcomes and advance Somaliland's progress toward global health targets, it is imperative to address the identified socioeconomic, healthcare, and geographic disparities in skin-to-skin contact.

## Methods

### Study setting

With a population of about 4.5 million, Somaliland is an independent nation in the Horn of Africa where the study was carried out. Geographically, Somaliland is located between Djibouti, Ethiopia, and Somalia. It is 176,119.2 km2, with 850 km of coastline. Its terrain is varied, ranging from hilly northern areas (1,800-2,100 m) to coastal plains. Wet and dry seasons alternate in the climate. The majority of the population is Muslim and of Somali ethnicity. More than one-third of the population lives in the capital, Hargeisa. Administratively, Somaliland is divided into 22 districts and six regions. Despite growing as a result of livestock exports, Somaliland's economy still faces difficulties because it is still a low-income nation with few opportunities for foreign investment and trade. In 2018, the Gulf States' estimated GDP of USD 2.5 billion and per capita GDP of USD 566 came from remittances and livestock exports. However, the country's access to bilateral agreements and direct donor investments is severely limited by its lack of international recognition.

### Study design, data source, and study period

The 2020 Somaliland Demographic Health Survey (SLHDS), the nation's first nationally representative health survey, provided the data used in the study. The 2020 SLHDS aimed to improve Somaliland's data systems and encourage evidence-based planning.

### Variables of the study

#### Outcome variable

The outcome variable was skin-to-skin contact (SSC) immediately after birth. Information on SSC was obtained from the 2020 Somaliland Demographic and Health Survey (SLDHS) women's questionnaire, in which mothers were asked whether their newborn had been placed directly on the mother's bare chest immediately after delivery. Responses were recorded as “Yes” or “No”. For the purpose of this study, SSC was analyzed as a binary outcome and coded as 1 = Yes (newborn placed immediately on the mother's bare chest after birth) and 0 = No (newborn not placed on the mother's bare chest immediately after birth).

#### Independent variables

Independent variables were grouped into individual-level and community-level factors. Individual-level variables included maternal age, education, wealth index, parity, age at first birth, birth order, antenatal care utilization, place of delivery, pregnancy wantedness, sex of the child, size of the child at birth, media exposure, husband's education, and husband's employment status. Community-level variables included region and place of residence (urban or rural). All study variables were obtained from the 2020 Somaliland Demographic and Health Survey (SLDHS). Skin-to-skin contact after birth was measured using mothers' responses to whether the newborn was placed on the mother's bare chest immediately after delivery and was categorized as “Yes” or “No”. Antenatal care (ANC) utilization was categorized into three groups: not utilized (no ANC visit), partially utilized (1–3 ANC visits), and adequately utilized (four or more ANC visits), based on the WHO-focused antenatal care recommendation applied during the survey period. Wealth index was classified into five quintiles (lowest, second, middle, fourth, and highest) using the DHS household wealth index classification. Media exposure was measured based on exposure to radio, television, or newspapers and categorized as “Yes” or “No.” The place of delivery was categorized as home or health institution delivery.

#### Data processing and analysis

This study used Stata version 17 for data processing and statistical analysis, while R software (RStudio) was used for spatial analysis. Before analysis, the dataset was assessed for missing and incomplete observations. Records with missing information on the outcome variable (skin-to-skin contact after birth) were excluded from the analysis. For explanatory variables, cases with incomplete information were handled using complete-case analysis to maintain consistency across the multivariable models. Descriptive statistics such as frequencies and percentages were used to summarize the study population. To account for the complex sampling design of the 2020 Somaliland Demographic and Health Survey (SLDHS), all analyses were weighted using the women's sampling weight. In addition, clustering and stratification were adjusted using the primary sampling unit (PSU) and strata variables to obtain nationally representative estimates and valid standard errors. Bivariate analysis was conducted using the chi-square test to examine the association between independent variables and skin-to-skin contact. Variables included in the multivariable analysis were selected based on theoretical relevance, evidence from previous literature, biological plausibility, and their importance in maternal and newborn health, rather than solely relying on statistical significance in the bivariate analysis. To account for the hierarchical structure of the data, where individuals are nested within clusters, a multilevel mixed-effects logistic regression model was employed, including four models: the null model, individual-level model, community-level model, and a final combined model. Adjusted Odds Ratios (AOR) with 95% Confidence Intervals (CI) were reported, and statistical significance was declared at *p* < 0.05. Model fitness was assessed using the Akaike Information Criterion (AIC), Bayesian Information Criterion (BIC), and log-likelihood values, while random effects were evaluated using the intra-class correlation coefficient (ICC) and tau-squared (*τ*^2^). For spatial analysis, regional shapefiles of Somaliland were obtained from publicly available geographic boundary data and imported into RStudio for mapping and spatial statistical analysis. The prevalence of skin-to-skin contact was aggregated at the regional level and linked to the shapefile using regional identifiers. For spatial analysis, regional shapefiles of Somaliland were obtained from publicly available geographic boundary data and imported into RStudio for mapping and spatial statistical analysis. The prevalence of skin-to-skin contact was aggregated at the regional level and linked to the shapefile using regional identifiers. Spatial visualization was performed using choropleth maps, while hotspot analysis was conducted using the Getis-Ord Gi statistic to identify areas with significantly high and low prevalence. Local Moran's I analysis was applied to assess spatial autocorrelation and clustering patterns across regions. Finally, the findings were interpreted in relation to existing literature to identify key determinants and inform evidence-based recommendations for improving maternal and newborn health practices. Multicollinearity among explanatory variables was assessed using the Variance Inflation Factor (VIF). The findings showed no evidence of serious multicollinearity among the independent variables, as all VIF values were within acceptable limits.

## Results

### Sociodemographic characteristics of the study participants

Table 1 presents the weighted socio-demographic and obstetric characteristics of the study participants. The majority of women were within the age group of 25–29 years (28.63%), followed by those aged 30–34 years (22.87%) and 20–24 years (16.74%), indicating that most participants were in their prime reproductive age. Regarding regional distribution, the largest proportions were from Marodijeh (26.75%) and Togdheer (25.89%), while Sahil accounted for the smallest share (5.50%). More than half of the participants resided in rural areas (55.31%), highlighting a predominantly rural population. In terms of educational attainment, a substantial majority of women had no formal education (78.64%), with only a small proportion attaining secondary (3.83%) or higher education (2.16%). Similarly, most husbands had no formal education (78.61%). The wealth distribution was relatively balanced across quintiles, although the highest (28.80%) and lowest (28.01%) categories represented the largest shares.

**Table 1:** Weighted distribution of socio-demographic characteristics of women in the study population.

Characteristic	Category	Weighted Frequency	Weighted Percentage (%)
Age	15–19	117.1	4.85
	20–24	403.8	16.74
	25–29	690.5	28.63
	30–34	551.5	22.87
	35–39	423.3	17.55
	40–44	175.6	7.28
	45–49	50.3	2.09
Region	Awdal	238.4	9.88
	Marodijeh	645.2	26.75
	Sahil	132.6	5.50
	Togdheer	624.5	25.89
	Sool	340.5	14.12
	Sanaag	430.8	17.86
Place of residence	Rural	1,334.0	55.31
	Urban	1,078.0	44.69
Education level	No education	1,896.7	78.64
	Primary	370.7	15.37
	Secondary	92.4	3.83
	Higher	52.1	2.16
Wealth index	Lowest	675.7	28.01
	Second	330.9	13.72
	Middle	261.0	10.82
	Fourth	449.7	18.65
	Highest	694.6	28.80
Parity	Primipara	303.5	12.58
	Multipara	1,052.5	43.63
	Grand multipara	1,056.1	43.78
Age at first birth	<20	1,070.6	44.38
	20–24	922.9	38.26
	25–30	362.8	15.04
	≥30	55.7	2.31
Husband attended school	No	1,896.1	78.61
	Yes	515.9	21.39
Husband worked last 12 months	Yes	1,195.6	49.57
	No	1,216.4	50.43
Birth order	1	298.8	12.39
	2	383.4	15.89
	≥3	1,729.9	71.72
Type of birth	Single	2,390.4	99.11
	Multiple	21.6	0.89
Sex of child	Male	1,307.5	54.21
	Female	1,104.4	45.79
Pregnancy wantedness	Then	1,645.5	68.22
	Later	631.8	26.20
	No more	134.6	5.58
ANC visits	Not utilized	1,270.6	52.68
	Partially utilized	687.2	28.49
	Adequately utilized	454.2	18.83
Place of delivery	Home	1,462.3	60.62
	Health institution	949.7	39.38
Size of child at birth	Very large	173.4	7.19
	Larger than average	180.6	7.49
	Average	1,547.7	64.17
	Smaller than average	122.4	5.08
	Very small	92.5	3.83
	Don't know	295.4	12.25
Media exposure	No	1,452.6	60.22
	Yes	959.4	39.78

From a reproductive perspective, most women were multiparous (43.63%) or grand multiparous (43.78%), and a large proportion had their first birth before the age of 20 (44.38%). Additionally, the majority of births were of higher order (≥3) (71.72%), reflecting high fertility patterns in the population. Almost all births were single (99.11%), and slightly more than half of the children were male (54.21%). Regarding maternal healthcare utilization, more than half of the women did not utilize antenatal care (ANC) services (52.68%), and a majority delivered at home (60.62%). Furthermore, most children were reported as having an average size at birth (64.17%). Notably, a considerable proportion of women had no exposure to media (60.22%), which may have implications for access to health information.

Overall, the findings suggest that the study population is characterized by low educational attainment, high fertility, limited access to maternal healthcare services, and a predominantly rural residence, all of which may influence maternal and child health outcomes.

### Bivariate analysis of factors associated with skin-to-skin contact after birth among women in Somaliland

Table 2 presented bivariate analysis showed that region, maternal education, birth order, wealth index, husband's employment status, husband's education, antenatal care utilization, place of delivery, size of child at birth, and media exposure were significantly associated with skin-to-skin contact after birth (*p* < 0.05). However, these findings represent crude associations only and should be interpreted cautiously. Variables identified from bivariate analysis, together with theoretically relevant variables, were further examined in the multilevel multivariable logistic regression model to identify independent predictors after adjusting for potential confounders.

**Table 2:** Bivariate analysis of factors associated with skin-to-skin contact after birth among women in the study population.

Variable	Category	skin-to-skin contact		*χ*²	*p*-value
		No (%)	Yes (%)		
Age	15–19	4.88	4.78	1.08	0.371
	20–24	16.78	16.64		
	25–29	27.13	32.09		
	30–34	22.39	23.97		
	35–39	18.81	14.64		
	40–44	7.62	6.50		
	45–49	2.39	1.37		
Region	Awdal	7.94	14.38	9.65	<0.001
	Marodijeh	24.35	32.28		
	Sahil	4.53	7.72		
	Togdheer	25.09	27.74		
	Sool	16.87	7.77		
	Sanaag	21.22	10.11		
Residence	Rural	55.26	55.40	0.00	0.970
	Urban	44.74	44.60		
Education level	No education	83.52	67.37	10.68	<0.001
	Primary	12.10	22.90		
	Secondary	2.68	6.49		
	Higher	1.70	3.23		
Birth order	1	11.25	15.00	4.88	0.008
	2	14.37	19.41		
	≥3	74.38	65.59		
Wealth index	Lowest	36.03	9.52	32.47	<0.001
	Second	15.37	9.91		
	Middle	10.19	12.29		
	Fourth	15.98	24.80		
	Highest	22.43	43.48		
Parity	Primipara	11.43	15.25	2.82	0.062
	Multipara	42.75	45.68		
	Grand multipara	45.83	39.08		
Age at first birth	<20	46.95	38.46	2.70	0.053
	20–24	37.09	40.97		
	25–30	13.96	17.54		
	≥30	2.00	3.02		
Type of birth	Single	98.95	99.48	0.79	0.374
	Multiple	1.05	0.52		
Husband employment	Yes	40.80	69.80	56.05	<0.001
	No	59.20	30.20		
Husband education	No	81.75	71.37	16.52	0.0001
	Yes	18.25	28.63		
ANC visits	Not utilized	63.16	28.52	44.48	<0.001
	Partially utilized	24.03	38.79		
	Adequately utilized	12.82	32.70		
Sex of child	Male	52.19	58.86	3.10	0.080
	Female	47.81	41.14		
Pregnancy wantedness	Then	69.90	64.35	2.76	0.065
	Later	24.24	30.70		
	No more	5.86	4.95		
Place of delivery	Home	75.66	25.95	297.86	<0.001
	Health institution	24.34	74.05		
Size of child at birth	Very large	5.58	10.89	5.11	0.0003
	Larger than average	6.20	10.46		
	Average	67.03	57.57		
	Smaller than average	4.04	7.48		
	Very small	3.45	4.72		
	Don't know	13.70	8.89		
Media exposure	No	64.73	49.83	20.46	<0.001
	Yes	35.27	50.17		

### Model comparison

Table 3 presents the results of the random effects analysis and model comparison across four models (Null Model, Model I, Model II, and Model III). The null model revealed substantial clustering of skin-to-skin contact across communities, as indicated by a relatively high intra-class correlation coefficient (ICC = 0.3289), suggesting that approximately 32.9% of the variation in the outcome was attributable to differences between clusters. The corresponding tau-squared (*τ*^2^ = 1.6125) further confirmed significant between-cluster variability. The likelihood ratio (LR) test was statistically significant (*p* < 0.001), supporting the use of a multilevel modeling approach. With the inclusion of individual-level variables in Model I, there was a marked reduction in both *τ*^2^ (0.0924) and ICC (0.0273), indicating that a considerable proportion of the between-cluster variation was explained by these variables. The improvement in model fit was also evident from the increase in log-likelihood (−1222.603) and the reduction in AIC (2,513.205) compared to the null model. The LR test remained statistically significant (*p* = 0.0028), confirming that Model I provided a better fit than the null model. Further adjustment with additional variables in Model II resulted in a continued decline in *τ*^2^ (0.0420) and ICC (0.0126), suggesting that more of the cluster-level variation was accounted for. The model fit improved further, as indicated by a higher log-likelihood (−1,140.317) and a lower AIC (2,302.634). However, the LR test became marginally non-significant (*p* = 0.0560), indicating diminishing returns in model improvement. In the final model (Model III), the between-cluster variance was substantially reduced (*τ*^2^ = 0.0091), with a minimal ICC (0.0028), indicating that almost all the variability in skin-to-skin contact was explained by the included variables. Model III demonstrated the best overall fit, as evidenced by the highest log-likelihood (−1,097.023) and the lowest AIC (2,280.047). Although the likelihood ratio (LR) test for Model III was not statistically significant (*p* = 0.3109), the model was retained as the final model because it demonstrated the best overall fit based on the lowest AIC and highest log-likelihood values. In addition, the inclusion of both individual- and community-level variables substantially reduced the between-cluster variance and intra-class correlation coefficient (ICC), indicating that most of the variability in skin-to-skin contact was explained by the final combined model. Therefore, Model III was considered the most parsimonious and theoretically appropriate model for explaining the outcome.

**Table 3:** Random effect analysis and model comparison.

Parameters	Null Model	Model I	Model II	Model III
Tau-squared (*τ*²)	1.6125	0.0924	0.0420	0.0091
ICC	0.3289	0.0273	0.0126	0.0028
Log-likelihood	−1,360.847	−1,222.603	−1,140.317	−1,097.023
AIC	2,725.694	2,513.205	2,302.634	2,280.047
BIC	2,737.408	2,712.346	2,367.062	2,531.902
LR test	173.42	7.69	2.53	0.24
*p*-value	<0.001	0.0028	0.0560	0.3109

ICC, Intra-class Correlation Coefficient; AIC, Akaike Information Criterion; BIC, Bayesian Information Criterion.

### Multilevel logistic regression analysis of factors associated with skin-to-skin contact after birth

Table 4 presents the multilevel mixed-effects logistic regression analysis of individual- and community-level factors associated with skin-to-skin contact after birth among women in Somaliland. In the final model (Model III), both individual- and community-level factors were independently associated with skin-to-skin contact practice.

**Table 4:** Multilevel mixed-effects logistic regression analysis of factors associated with skin-to-skin contact after birth among women in Somaliland.

Variable	Null model	Model I	Model II	Model III
		AOR with 95%CI	AOR with 95%CI	AOR with 95%CI
Age				
15–19		Ref	Ref	Ref
20–24		0.87 (0.47–1.59)		0.86 (0.45–1.63)
25–29		0.92 (0.51–1.65)		0.86 (0.45–1.63)
30–34		0.85 (0.43–1.68)		0.87 (0.43–1.77)
35–39		0.85 (0.43–1.68)		0.80 (0.35–1.84)
40–44		0.72 (0.28–1.8)		0.62 (0.24–1.56)
45–49		0.41 (0.11–1.53)		0.55 (0.17–1.77)
Region				
Awdal		Ref	Ref	Ref
Marodijeh			0.46 (0.31–0.68) ***	0.36 (0.23–0.56) ***
Sahil			1.03 (0.74–1.43)	1.05 (0.71–1.55)
Togdheer			0.70 (0.47–1.04)	1.05 (0.71–1.55) *
Sool			0.50 (0.38–0.66) ***	0.48 (0.34–0.68) ***
Sanaag			0.57 (0.39–0.84) **	0.54 (0.34–0.86) **
Residence				
Rural	Ref	Ref	Ref	Ref
Urban			1.20 (0.92–1.56)	1.14 (0.84–1.53)
Education level				
No education	Ref	Ref	Ref	Ref
Primary		1.21 (0.75–1.94)		1.20 (0.68–2.11)
Secondary		1.12 (0.58–2.13)		0.91 (0.46–1.81)
Higher		0.64 (0.23–1.77)		0.52 (0.18–1.49)
Birth order				
1		Ref		
2		2.01 (0.32–12.50)		2.96 (0.62–14.25)
≥3		1.77 (.26–11.8)		3.32 (0.63–17.46)
Wealth index				
Lowest	Ref	Ref	Ref	Ref
Second		2.15 (1.57–2.94) ***		1.91 (1.38–2.64) ***
Middle		3.10 (2.13–4.53) ***		2.07 (1.34–3.19) **
Fourth		3.66 (2.68–4.99) ***		1.95 (1.31–2.90) **
Highest		3.39 (2.30–4.98) ***		1.64 (1.09–2.46) *
Parity				
Primipara	Ref	Ref	Ref	Ref
Multipara		0.98 (0.59–1.61)		0.38 (0.07–2.02)
Grand multipara		1.04 (0.61–1.76)		0.39 (0.08–1.90)
Age at first birth				
<20	Ref	Ref	Ref	Ref
20–24		1.22 (0.93–1.60)		1.14 (0.84–1.55)
25–30		1.58 (1.14–2.18) *		1.43 (0.97–2.10)
≥30		2.07 (0.81–5.27)		2.34 (0.87–6.27)
Type of birth				
Single	Ref	Ref	Ref	Ref
Multiple		0.359 (0.062–2.09)		0.226 (0.043–1.205)
Husband employment				
Yes	Ref	Ref	Ref	Ref
No			0.55 (0.38–0.80) **	0.70 (0.48–1.02)
Husband education				
No	Ref	Ref	Ref	Ref
Yes			0.78 (0.56–1.09)	0.71 (0.50–1.01) *
ANC visits				
Not utilized	Ref	Ref	Ref	Ref
Partially utilized		2.43 (1.71–3.44) ***		1.53 (1.01–2.33) *
Adequately utilized		3.59 (2.39–5.40) ***		1.79 (1.13–2.85) *
Sex of child				
Male	Ref	Ref	Ref	Ref
Female		0.73 (0.56–0.94) *		0.79 (0.61–1.03)
Pregnancy wantedness				
Then	Ref	Ref	Ref	Ref
Later		1.50 (1.10–2.03) *		1.47 (1.13–1.91) **
No more		1.56 (0.91–2.68)		1.47 (1.13–1.91)
Place of delivery				
Home	Ref	Ref	Ref	Ref
Health institution			8.02 (6.08–10.58) ***	6.09 (4.39–8.45) ***
Size of child at birth				
Very large	Ref	Ref	Ref	Ref
Larger than average		0.73 (0.36–1.49)		0.66 (0.29–1.50)
Average		0.51 (0.296-.89)		0.53 (0.29–0.99) *
Smaller than average		0.82 (0.34–1.95)		0.53 (0.29–0.99)
Very small		0.91 (0.32–2.57)		1.11 (0.45–2.73)
Don't know		0.53 (0.27–1.05)		0.88 (0.28–2.70)
Media exposure				
No	Ref	Ref	Ref	Ref
Yes		1.53(1.17–2.01) **		0.55(0.25–1.21) **

AOR, Adjusted Odds Ratio; Ref, Reference category.

* *p* < 0.05.

** *p* < 0.01.

*** *p* < 0.001.

At the community level, region remained a significant predictor of skin-to-skin contact after adjusting for other covariates. Women residing in Marodijeh (AOR = 0.36; 95% CI: 0.23–0.56), Sool (AOR = 0.48; 95% CI: 0.34–0.68), and Sanaag (AOR = 0.54; 95% CI: 0.34–0.86) had significantly lower odds of practicing skin-to-skin contact compared with women residing in Awdal. However, place of residence was not significantly associated with skin-to-skin contact in the adjusted model.

At the individual level, wealth index, antenatal care utilization, place of delivery, pregnancy wantedness, and size of child at birth were significantly associated with skin-to-skin contact practice. Women from wealthier households had higher odds of practicing skin-to-skin contact compared with women from the lowest wealth quintile. Similarly, women who partially utilized ANC services (AOR = 1.53; 95% CI: 1.01–2.33) and those who adequately utilized ANC services (AOR = 1.79; 95% CI: 1.13–2.85) were more likely to practice skin-to-skin contact compared with women who did not utilize ANC services.

Place of delivery was the strongest predictor of skin-to-skin contact practice. Women who delivered in health institutions had significantly higher odds of practicing skin-to-skin contact (AOR = 6.09; 95% CI: 4.39–8.45) compared with women who delivered at home. In addition, women who reported that the pregnancy was wanted later had higher odds of practicing skin-to-skin contact compared with women whose pregnancy was wanted at that time. Child size at birth was also significantly associated with skin-to-skin contact.

Maternal age, maternal education, parity, age at first birth, type of birth, husband's employment status, and sex of the child were not significantly associated with skin-to-skin contact in the final adjusted model.

### Spatial analysis

**Figure 1:**
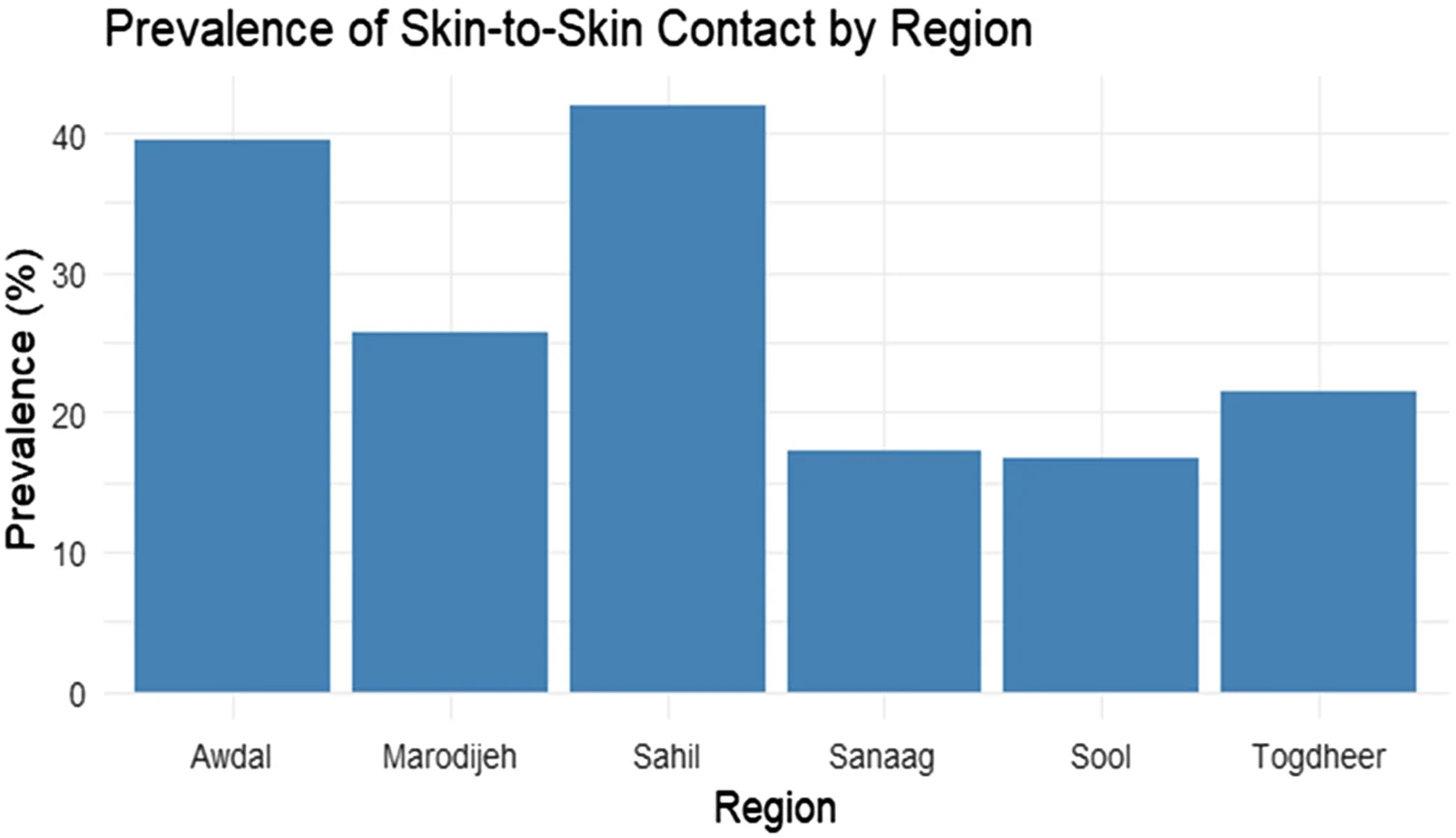
Prevalence of skin-to-skin contact after birth across regions in Somaliland.

Figure 1 illustrates the prevalence of skin-to-skin contact after birth across regions in Somaliland. The findings show marked regional variation in the practice. The highest prevalence was observed in Sahil (approximately 42%), followed by Awdal (around 40%), indicating relatively better uptake in these regions. In contrast, the lowest prevalence was reported in Sanaag (approximately 17%) and Sool (around 16%–17%), suggesting limited practice in the eastern regions. Marodijeh (approximately 26%) and Togdheer (around 21%–22%) demonstrated moderate levels of prevalence. Overall, the distribution indicates a clear disparity across regions, with western regions showing higher levels of skin-to-skin contact compared to central and eastern regions. This variation may reflect differences in access to maternal healthcare services, health facility availability, and awareness of recommended newborn care practices.

**Figure 2:**
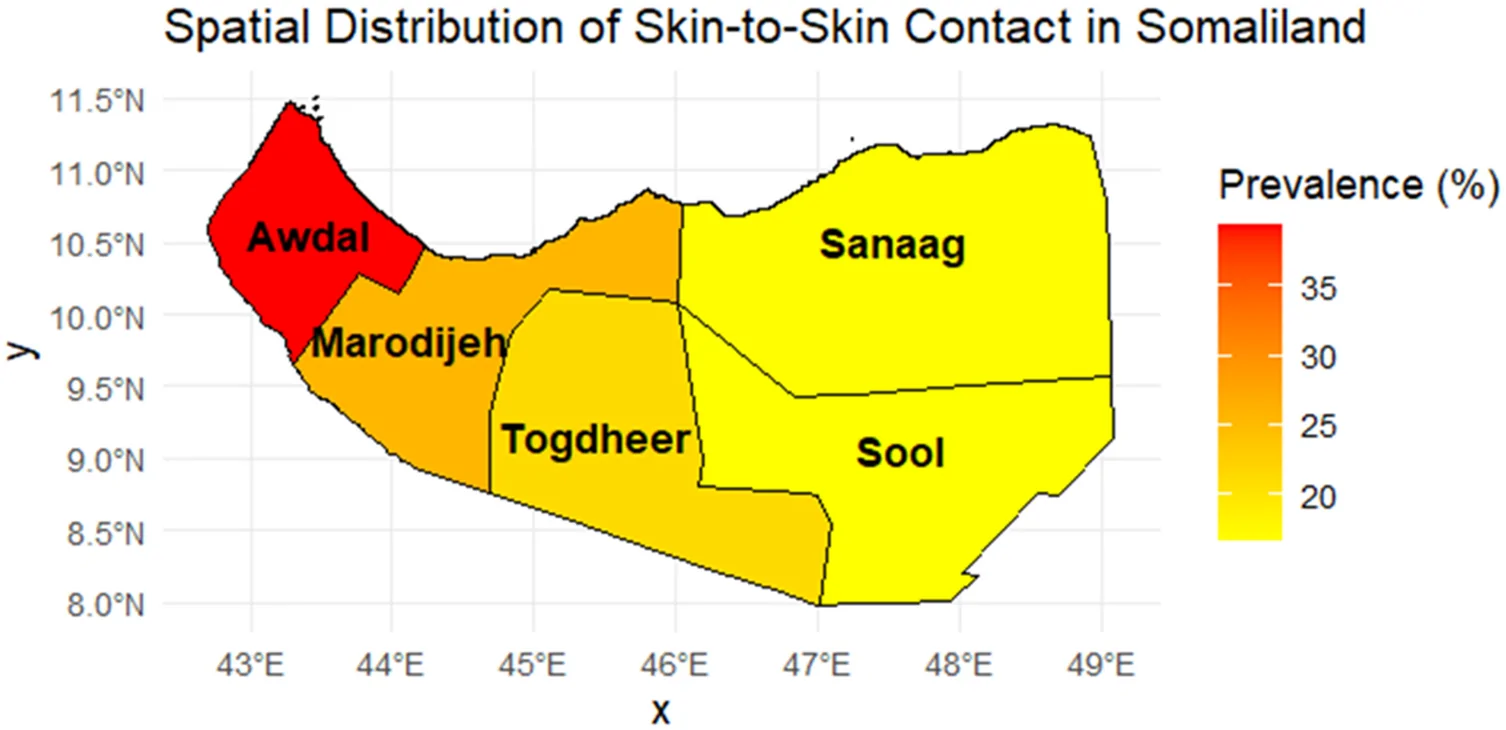
Spatial distribution of skin-to-skin contact after birth in Somaliland.

Figure 2 presents the spatial distribution of skin-to-skin contact after birth across Somaliland, revealing clear geographic disparities in the practice. The map shows a higher concentration of skin-to-skin contact in the western part of the country, particularly in Awdal, which exhibits the highest prevalence. Marodijeh and Togdheer display moderate levels, forming a transitional zone between high and low prevalence areas. In contrast, the eastern regions, including Sanaag and Sool, demonstrate consistently lower prevalence levels, as indicated by the lighter color gradients. This pattern suggests a distinct west-to-east decline in the practice of skin-to-skin contact. Overall, the spatial distribution indicates that the practice is not evenly distributed across the country but follows a clear geographic gradient. These differences may be attributed to variations in access to healthcare services, distribution of health facilities, and regional differences in awareness and implementation of recommended maternal and newborn care practices.

**Figure 3:**
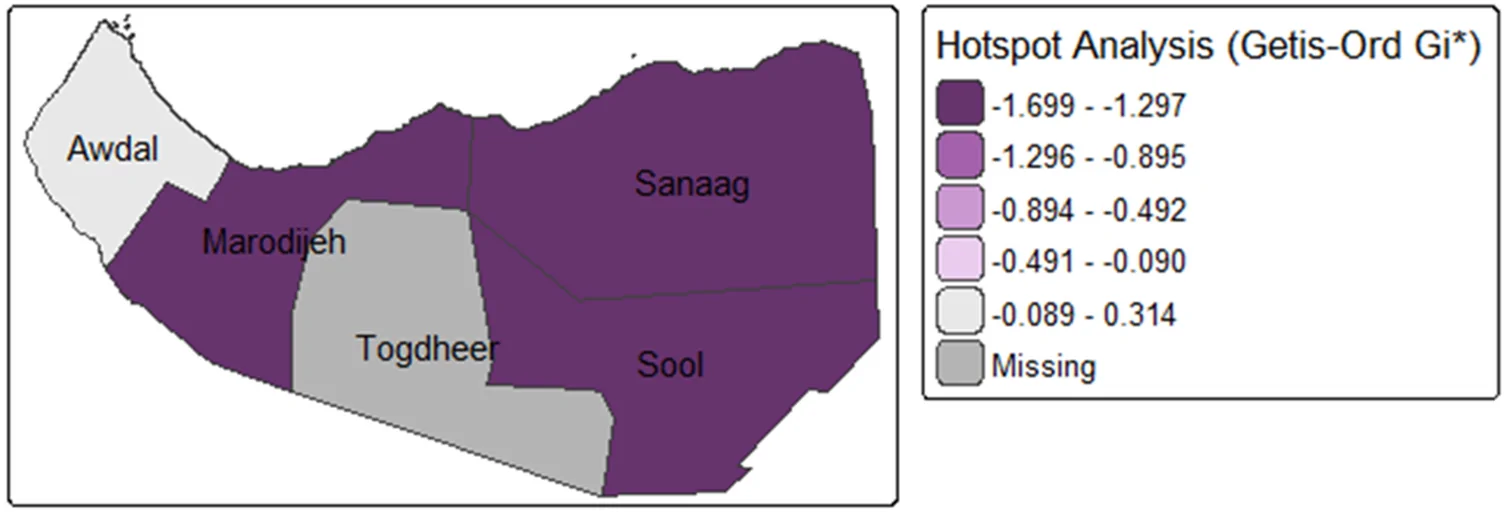
Hotspot analysis (getis-Ord Gi*) of skin-to-skin contact after birth in Somaliland.

Figure 3 presents the hotspot analysis of skin-to-skin contact using the Getis-Ord Gi* statistic, highlighting areas of significant spatial clustering. The results indicate the presence of cold spots (areas with significantly low prevalence) in the eastern regions of Somaliland, particularly in Sanaag and Sool, where consistently lower values of skin-to-skin contact are observed. Marodijeh also demonstrates relatively low clustering, suggesting suboptimal practice in this region. In contrast, Awdal appears to have relatively better performance; however, it does not exhibit a strong statistically significant hotspot cluster, indicating that high-prevalence areas are less spatially concentrated compared to low-prevalence regions. Togdheer shows no clear clustering pattern, suggesting a more neutral or transitional zone. Overall, the hotspot analysis reveals that low levels of skin-to-skin contact are geographically clustered, particularly in the eastern part of the country, emphasizing these areas as priority regions for targeted maternal and newborn health interventions.

**Figure 4:**
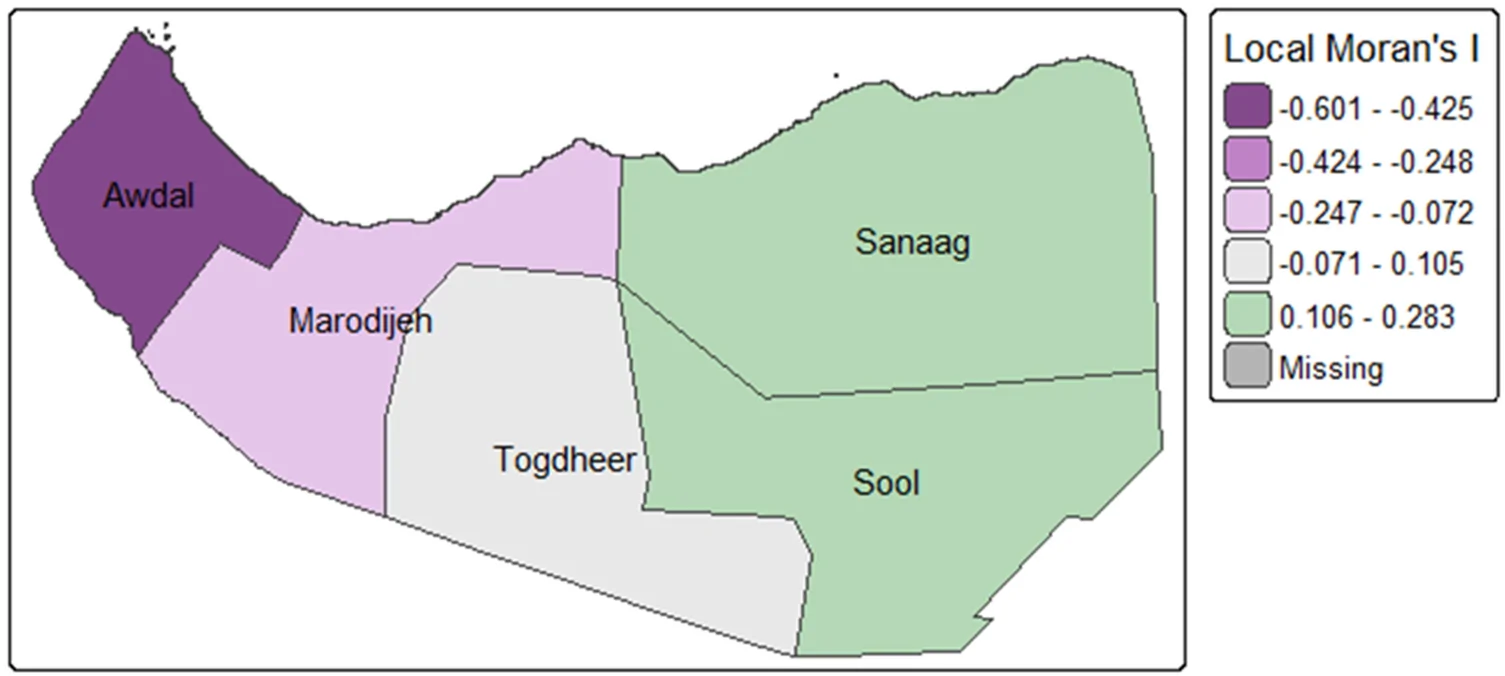
Local Moran's I analysis of skin-to-skin contact after birth in Somaliland.

Figure 4 presents the Local Moran's I analysis, illustrating the spatial autocorrelation of skin-to-skin contact across regions in Somaliland. The results indicate the presence of both positive and negative spatial clustering patterns. Sanaag and Sool exhibit positive spatial autocorrelation, suggesting that these regions are surrounded by neighboring areas with similarly low levels of skin-to-skin contact, forming clusters of low prevalence. In contrast, Awdal shows negative spatial autocorrelation, indicating that it differs from its neighboring regions by having relatively higher prevalence compared to surrounding areas. Marodijeh demonstrates moderate negative spatial association, suggesting some degree of spatial transition between high and low prevalence regions. Togdheer appears to have no significant spatial autocorrelation, indicating a more neutral or random distribution in this area. Overall, the Local Moran's I analysis confirms that the distribution of skin-to-skin contact is not random but spatially structured, with clear clustering of similar values across geographic regions. This highlights the importance of location-specific interventions to address regional inequalities in maternal and newborn care practices.

## Discussion

This study assessed the prevalence and determinants of skin-to-skin contact after birth among women in Somaliland using nationally representative data and advanced analytical approaches. The findings revealed that only 24.81% of newborns were placed on their mother's chest and bare skin immediately after birth, while the majority (75.19%) did not receive skin-to-skin contact. When comparing the findings of this study with evidence from other regions, the prevalence of skin-to-skin contact observed in Somaliland (24.81%) is higher than that reported in some South Asian countries such as India (14.5%) and Nepal (16.5%), but remains substantially lower than that reported in Pakistan (55%) and Sri Lanka (50%) (15, 16). Similarly, evidence from Sub-Saharan Africa demonstrates wide variation in the practice of skin-to-skin contact. A pooled analysis of Demographic and Health Survey data from 17 countries reported the lowest prevalence in Nigeria (11%) and the highest in Benin (75.1%) (14). These variations across countries and regions may reflect differences in healthcare system capacity, coverage of skilled birth attendance, cultural practices, and the implementation of maternal and newborn health programs. The relatively moderate prevalence observed in this study suggests that, although some progress has been made, significant gaps remain in the adoption of recommended newborn care practices in Somaliland.

Maternal healthcare utilization was recognized as a principal factor influencing skin-to-skin contact. Women who engaged in antenatal care (ANC), whether partially or sufficiently, exhibited a significantly higher propensity for practicing skin-to-skin contact in comparison to those who did not utilize ANC services. This finding aligns with prior research that emphasizes ANC as a crucial medium for advancing early newborn care practices via health education and counselling (17, 18). Frequent interactions with healthcare providers during ANC visits increase maternal awareness and elevate the probability of adopting recommended practices. The place of delivery proved to be the most significant predictor of skin-to-skin contact. Women who gave birth in health facilities were considerably more inclined to engage in skin-to-skin contact than those who delivered at home. This is consistent with findings from studies conducted in Ethiopia and other low-income nations, which indicate that facility-based delivery significantly enhances compliance with essential newborn care practices owing to the availability of skilled birth attendants (14, 19). On the other hand, home deliveries often don't have professional help, which means that people don't follow the recommended practices as much.

Socioeconomic status was also very important. Women from wealthier households were more likely to engage in skin-to-skin contact than those from less affluent backgrounds. This finding illustrates broader disparities in access to healthcare services, as affluent households are more capable of surmounting financial and logistical obstacles to care. Similar finding was reported in a related study in Nigeria (20). Studies have shown that mothers in higher wealth index are more likely to exhibit better health seeking behaviors, including having institutional births and attending more ANC follow-up visits (21, 22), increasing their knowledge of the advantages of skin-to-skin contact.

The spatial analysis revealed marked geographic disparities in the practice of skin-to-skin contact. A clear west-to-east gradient was observed, with higher prevalence in western regions such as Awdal and Sahil, and lower prevalence in eastern regions including Sanaag and Sool. The hotspot and Local Moran's I analyses confirmed significant clustering of low practice in the eastern regions. These findings suggest that geographic location plays a critical role in shaping maternal health behaviors, likely due to differences in healthcare infrastructure, availability of skilled providers, and regional disparities in health service delivery. Similar spatial inequalities in maternal and newborn health practices have been reported in other African settings (18, 23). The findings of this study are consistent with recent evidence highlighting the importance of maternal healthcare utilization and facility-based delivery in promoting skin-to-skin contact and essential newborn care practices. Previous studies have demonstrated that access to skilled healthcare providers, antenatal counselling, and institutional delivery significantly improve adherence to recommended newborn care interventions, particularly in low- and middle-income countries. Furthermore, socioeconomic and geographic inequalities continue to influence maternal and neonatal healthcare utilization across many settings, contributing to disparities in early newborn care practices. Similar patterns of regional and healthcare-related inequalities have been reported in recent studies conducted in diverse low-resource settings (24–26).

Overall, the findings highlight that the practice of skin-to-skin contact in Somaliland remains suboptimal, with less than one-quarter of newborns receiving this essential care. Strengthening maternal healthcare services, particularly antenatal care and facility-based delivery, along with targeted, region-specific interventions, is essential to improve the uptake of skin-to-skin contact. Addressing both socioeconomic and geographic inequalities will be critical for enhancing neonatal care and reducing neonatal morbidity and mortality.

Despite its strengths, this study has several limitations that should be acknowledged. First, the cross-sectional nature of the SLDHS data limits the ability to establish causal relationships between the explanatory variables and skin-to-skin contact practices. Second, the study relied on self-reported information, which may be subject to recall bias and social desirability bias, particularly for maternal and newborn care practices occurring several years before the survey. Third, although the analysis adjusted for a range of individual- and community-level factors, residual confounding from unmeasured variables such as quality of healthcare services, provider competency, cultural beliefs, and health system readiness could not be fully ruled out. In addition, the use of secondary survey data restricted the analysis to variables available in the dataset. Nevertheless, the study provides nationally representative evidence and valuable insights into the socioeconomic and geographic inequalities in skin-to-skin contact practices in Somaliland.

## Conclusion

This study revealed that the prevalence of skin-to-skin contact after birth in Somaliland remains suboptimal, with only 24.81% of newborns placed on their mother's chest and bare skin immediately after delivery. This indicates that a large proportion of mothers and newborns are missing a critical, evidence-based intervention essential for improving neonatal survival and health outcomes. The findings demonstrated that both individual- and community-level factors significantly influence the practice of skin-to-skin contact. In particular, maternal healthcare utilization especially antenatal care attendance and place of delivery emerged as key determinants, with institutional delivery being the strongest predictor. Socioeconomic status also played an important role, highlighting persistent inequalities in access to maternal and newborn healthcare services. Furthermore, the spatial analysis revealed clear geographic disparities, with lower prevalence concentrated in specific regions, indicating that access to and uptake of essential newborn care practices are unevenly distributed across Somaliland.

These findings underscore the urgent need to strengthen maternal and newborn healthcare services. Interventions aimed at increasing antenatal care utilization, promoting facility-based delivery, and improving access to skilled healthcare providers are critical to enhancing the uptake of skin-to-skin contact. In addition, targeted, region-specific strategies are required to address the identified geographic inequalities, particularly in underserved areas.

### Policy implication

The findings of this study have important policy implications for improving maternal and newborn health in Somaliland. Strengthening antenatal care services by integrating education on essential newborn care, including skin-to-skin contact, is crucial to improve maternal awareness and practice. Promoting facility-based delivery should be prioritized, given its strong association with the uptake of skin-to-skin contact, through improved access, quality of care, and reduction of financial and geographic barriers. In addition, targeted, region-specific interventions are needed to address the observed geographic disparities, particularly in underserved areas. Community-based health education programs should also be expanded to increase awareness, especially in rural settings where home deliveries are common. Overall, integrating skin-to-skin contact into national maternal and newborn health strategies and strengthening health system implementation are essential to enhance coverage and improve neonatal outcomes.

### Strengths and limitations

This study has several notable strengths. It utilized nationally representative data, enhancing the generalizability of the findings to the wider population of women in Somaliland. The application of multilevel mixed-effects modeling allowed for the simultaneous assessment of individual- and community-level factors, providing more robust and reliable estimates. Additionally, the inclusion of spatial analysis offered deeper insights into geographic disparities in skin-to-skin contact, strengthening the overall analytical approach. However, the study also has limitations. The cross-sectional design limits the ability to establish causal relationships between variables. The use of self-reported data may introduce recall and reporting biases. Furthermore, the analysis was restricted to variables available in the survey dataset, and therefore could not account for factors such as quality of care, provider practices, and health system readiness. Despite these limitations, the study provides valuable evidence to inform maternal and newborn health interventions.

## Data Availability

Publicly available datasets were analyzed in this study. This data can be found here: The data used in this study are from the Somalia Demographic and Health Survey (SDHS 2020), a nationally representative dataset collected by the DHS Program. The dataset is not publicly available for direct download but can be accessed upon reasonable request and registration through the DHS Program (https://dhsprogram.com/). Researchers must obtain authorization from the DHS Program before accessing the data. The authors did not have any special access privileges that others would not have.
